# Effect of Multidirectional Isothermal Forging on Microstructure and Mechanical Properties in Ti-6Al-4V Alloy

**DOI:** 10.3390/ma15093156

**Published:** 2022-04-27

**Authors:** Zhichao Xu, Wenju Yang, Jianfeng Fan, Tao Wu, Zeng Gao

**Affiliations:** 1 School of Materials Science and Engineering, Henan Polytechnic University, Jiaozuo 454003, China; xzc@hpu.edu.cn (Z.X.); 15538306901@163.com (W.Y.); cl2020wutao@163.com (T.W.); 2 Key Laboratory of Interface Science and Engineering in Advanced Materials, Ministry of Education, Taiyuan University of Technology, Taiyuan 030024, China; fanjianfeng7@hotmaill.com

**Keywords:** Ti-6Al-4V alloy, multidirectional isothermal forging, microstructure, mechanical properties

## Abstract

In the present work, the microstructure and mechanical properties of Ti-6Al-4V alloy during multidirectional isothermal forging (MDIF) were systematically investigated. The evolution of the microstructure and texture of Ti-6Al-4V alloy during MDIF was studied using TEM and electron backscattered diffraction (EBSD). The experiment results showed that the grain size decreased with the increase in cumulative strain, especially in the easy deformation zone. After four deformation cycles, a homogeneous equiaxed grained microstructure with an average grain size of 0.14 μm was achieved. The texture changes of the alloy were studied in detail. After one cycle of MDIF, the texture was mainly composed of (0002) [01 10], and the Euler angles were (8°, 30°, 30°). The density of texture decreased with the increase in loading cycle, but the dispersion of texture increased. After four cycles of MDIF, the non-basal texture (1010) <1102> texture was observed, and the Euler angles were (82°, 33°, 0°). The highest achieved mechanical properties for Ti-6Al-4V alloy in the MDIF condition were a yield strength 900 MPa, ultimate tensile strength of 921 MPa, and an elongation of 12.1% at room temperature. The increase in MDIF cycles improved the hardness of the alloy. The significant improvement in mechanical properties was attributed to the ultrafine-grained microstructure.

## 1. Introduction

Ti and titanium alloys are characterized by their favorable comprehensive properties such as high specific strength, low density, good corrosion resistance, and unique features such as shape memory, superconductivity, hydrogen storage, and biocompatibility [1]. They have been widely used in modern fields such as mechanical engineering, instrument manufacturing, aerospace, and the medical industry [2]. Among the duplex α + β titanium alloys, the Ti-6Al-4V (TC4) alloy is the most commonly used, which accounts for 60% of industrial titanium [3]. As well known, microstructure has an important influence on the properties of the alloy. The Hall–Petch equation [4] shows that, when the grain size is reduced, the strength of the material at room temperature increases substantially. How to control and optimize the microstructure is significant in achieving desired mechanical properties. Ever since severe plastic deformation (SPD) was demonstrated as a practical approach to produce ultrafine-grained (UFG) metals [5], extensive research has been carried out to develop SPD techniques [6]. Several SPD processing techniques have been developed and reported, such as equal-channel angular pressing (ECAP) [7], high-pressure torsion (HPT) [8], multidirectional isothermal forging (MDIF) [9], and accumulative roll bonding (ARB) [10]. In recent years, multidirectional isothermal forging (MDIF) has been successfully applied for grain refinement and performance enhancement of magnesium [11], Ti-6Al-4V [12], Ti-48Al-2Nb-2Cr [13], and other alloys [14,15], since it has a simple process and low manufacturing cost.

Ansarian et al. [16] studied the microstructure evolution and mechanical properties of pure Ti under MDIF; the results showed that the pure Ti exhibited grain refinement, as well as high yield tensile strength and shear strength, attributed to twin crystal and dynamic recrystallization. Zhang [17] discovered that the MDIF process could enhance the mechanical properties of TC4 alloy, and the main reason was attributed to ultrafine grains. The mechanism of grain refinement during MDIF included continuous dynamic recrystallization (CDRX) and discontinuous dynamic recrystallization (DDRX). Salishchev [18] indicated that spheroidization and dynamic recrystallization lead to a significant refinement of the initial coarse grain during the MDIF process. Although many studies have focused on the MDIF process of TC4 alloy, the texture details of TC4 alloy during the MDIF process have rarely been reported. Optimizing the homogeneity of TC4 alloy has become a hot topic in recent years [19]. It is essential to establish the relationship between thermomechanical processing parameters and the texture evolution law of TC4 alloy, which is conducive to controlling mechanical properties.

In this paper, we studied the effects of the MDIF process on the microstructures and mechanical properties of TC4 alloy. The recrystallization mechanism, grain growth, and texture were evaluated using the EBSD and TEM techniques. This work could contribute to developing wide industrial applications of this promising material.

## 2. Materials and Methods

The as-received material used in the study was a commercially produced TC4 alloy. The chemical composition of the alloy is shown in Table 1. The transition temperature of β-phase in this alloy was reported to be ~975 °C [20].

**Figure 1:**
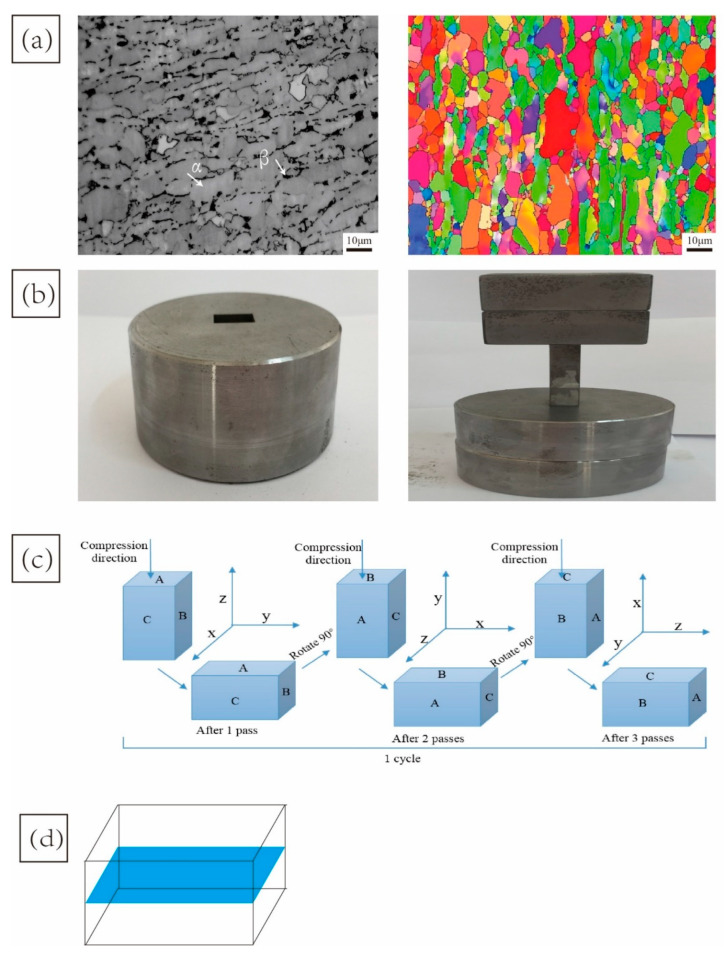
Initial structure of TC4 alloy and schematics showing the die and the coordinate systems: (**a**) initial structure of TC4 alloy; (**b**) MDIF die; (**c**) MDIF process diagram; (**d**) sampling position diagram.

This study chose TC4 alloy with equiaxed α organization and coarse β organization, as shown in Figure 1a. The black area represents the β-phase, while the gray area represents the α-phase. The phase composition is dominated by the α-phase, surrounded by a small amount of β-phase. The multidirectional compression mold, a nickel-based alloy, is shown in Figure 1b, with an internal cavity size of 10 mm × 15 mm × 35 mm. The specific machining process of MDIF is shown in Figure 1c. Before the subsequent forging, the pancake produced in the preceding step was canted through cutting the curved faces off the billet and rotating the pancake by 90°. In this article, every three passes constitute a cycle. The samples were deformed at 600 °C with a 33% height reduction at a strain rate of 10^−1^ s in each pass deformation. A graphite-based lubricant was used to reduce the friction between the tool and the sample. As shown in Figure 1d, the samples for microstructural examination after the first step and the second step of isothermal forging were taken from the middle area of the leftover materials. This is because the middle area is representative and convenient for comparison.

The microstructure was examined using optical microscopy (OM, Carl Zeiss, Axio Observer, Jena, Germany). Scanning electron microscopy (SEM) was performed with electron backscatter diffraction (EBSD) using a “TESCAN MIRA 3 LMH” (TESCAN, Brno, Czech Republic) microscope equipped with a field-emission gun and “Oxford Instruments HKL Channel 5” (Oxford Instruments, Abingdon, UK) system. Transmission electron microscopy (TEM) was performed using the “JEOL-2000EX” (JEOL, Tokyo, Japan) microscope. The TEM and SEM-EBSD examination objects were prepared by electropolishing in the “Tenupol-5” device. In this work, the β-phase was too difficult to accurately index; thus, the EBSD data, including grain diameter distribution charts, misorientation angle distribution charts, and pole figures, were analyzed only for the α-phase. The HV-1000MD Vickers hardness tester (Light-Mach Tech. Co., Ltd., Shanghai, China) was used to measure the microhardness of different areas in the joints, and the interval between adjacent test points was 0.25 mm. The test load was 25 g with a dwell time of 10 s. Tensile tests were conducted on the Zwick Z100 servohydraulic testing system (Zwick Roell, Ulm, Germany) at an initial strain rate of 10^−3^ s^−1^.

## 3. Results and Discussion

### 3.1. Microstructure of Samples after One Cycle of MDIF

**Figure 2:**
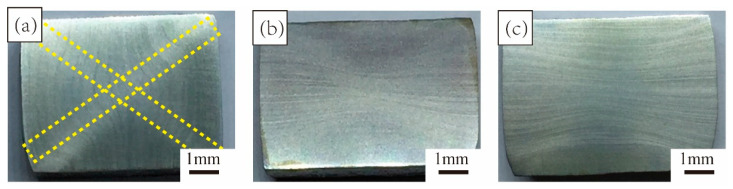
Microstructure of TC4 alloy after one cycle of MDIF: (**a**) one pass; (**b**) two passes; (**c**) three passes.

Figure 2 shows the microstructure of the TC4 alloy after one cycle of MDIF. It can be seen that, after the first deformation, the surface of the specimen had a brighter and “X” shaped macroscopic band than the matrix, which is a typical unidirectional compression deformation characteristic. With the continuous transformation of the loading axis, the microstructure was elongated along the shear direction.

**Figure 3:**
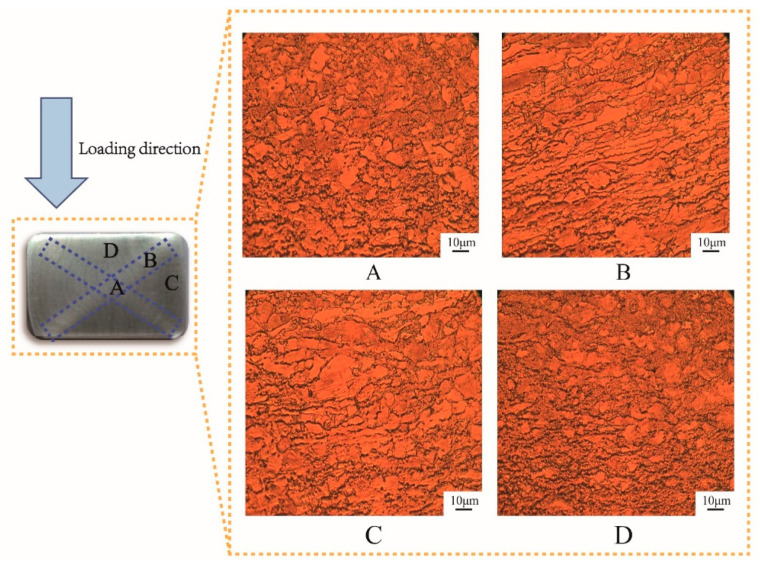
Microstructure of TC4 alloy in different zones after one cycle of MDIF: (**A**) central area; (**B**) shear region; (**C**) cross region I; (**D**) cross region II.

In order to better illustrate the microstructure evolution under one cycle of MDIF, we analyzed the “X”-shaped macroscopic zone. As can be seen in Figure 3, the microstructure of each part showed a significant difference after one cycle of MDIF. The C and D areas were in the region where the two shear directions cross. The microstructure in this area was more uniform, and the grain morphology was mainly equiaxed. In addition, a banded structure appeared along the shear direction. Area B was located at an angle of 45° to the loading direction, and the microstructure of the alloy consisted of banded grains. Area A was in the center of the sample and had a triaxial compressive stress state. Due to the generation of deformation heat, this area was the best area for deformation development. The deformation zone was distributed perpendicular to the compression direction. One can notice that the microstructure could be divided into several units by the deformation zone. In this area, the microstructure of the alloy was characterized by predominantly refined grains. After one cycle of MDIF, the average grain size of the α-phase was refined from 4.8 μm to 0.68 μm.

**Figure 4:**
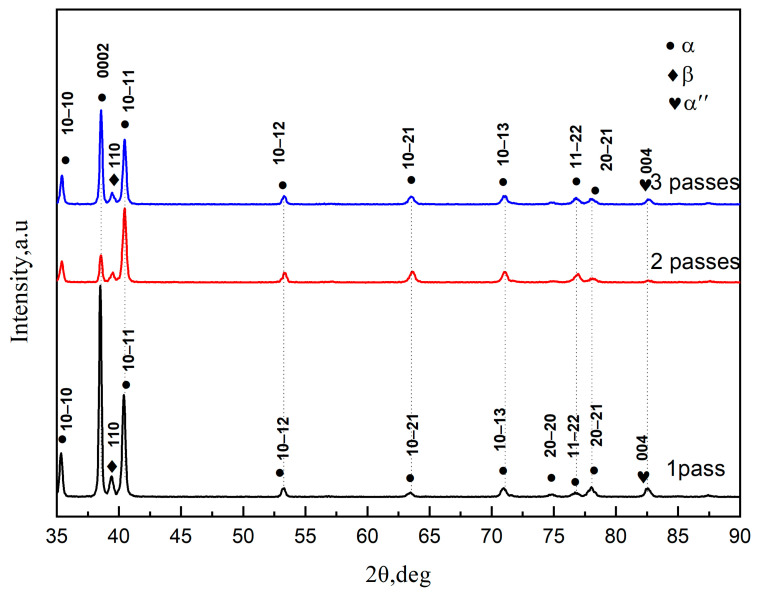
XRD pattern of TC4 alloy during each pass processed by MDIF.

Figure 4 shows the XRD patterns of the TC4 alloy processed by MDIF at different passes. Three phases appeared in the alloys: α-phase, β-phase, and α″-phase. With increasing MDIF passes, the intensity of the (0002), (1010), and (1011) crystal plane diffraction peaks decreased.

**Figure 5:**
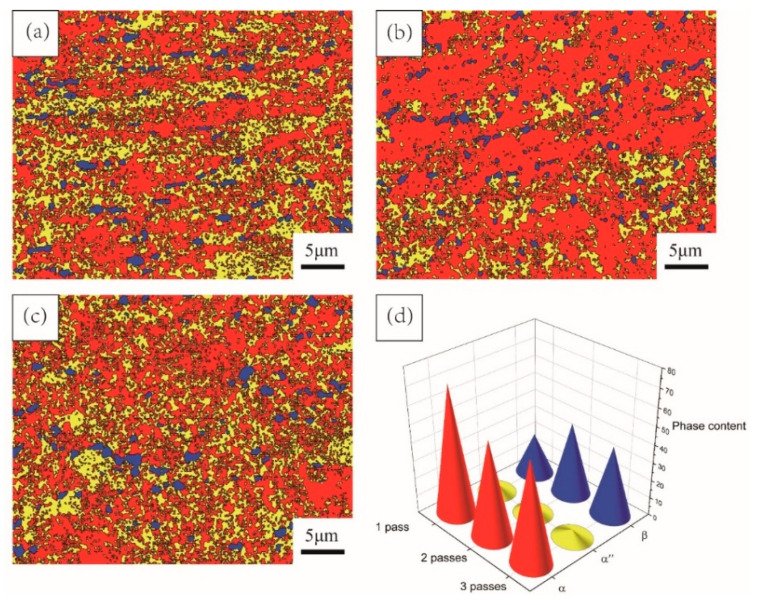
Phase distribution of TC4 alloy after one cycle of MDIF: (**a**) one pass; (**b**) two passes; (**c**) three passes; (**d**) statistical chart.

To obtain more information about the phase distribution of the alloy, an EBSD study was conducted. Figure 5 shows the phase distribution of TC4 alloy after one cycle of MDIF. The α-phase is represented by red, the β-phase is represented by blue, and the α″-phase is represented by yellow. As shown in Figure 5a–c, the β-phase was distributed along the deformation zone. With the increase in cumulative deformation, the uniformity of the β-phase distribution worsened. On the contrary, the distribution of the α″-phase gradually became more uniform. According to the statistical data offered by channel 5, it can be concluded that the content of β-phase and α″-phase increased and then decreased with the increase in accumulated deformation, while the content of α-phase decreased and then increased, as shown in Figure 5d.

**Figure 6:**
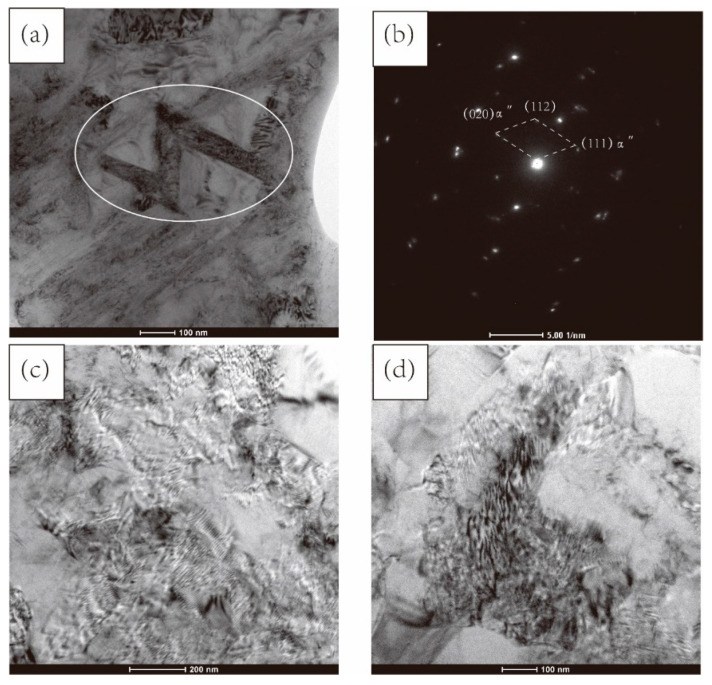
TEM image of TC4 alloy after one cycle of MDIF: (**a**,**b**) one pass; (**c**) two passes; (**d**) three passes.

Figure 6 presents a TEM analysis of TC4 alloy processed by one cycle of MDIF. After one pass of MDIF, the dislocation density was high, and dislocations were heavily concentrated at the grain boundaries. The TEM image shows that a lamellar structure (marked by white line) appeared in the matrix. The fast Fourier transform (FFT) pattern corresponding to the white frame is shown in Figure 6b, which illustrates that the corresponding phase was the α″-phase with an orthorhombic martensite crystal structure. With the progress of deformation, the density of dislocations decreased, attributed to the mutual entanglement and annihilation of dislocations, and many cellular substructures could be observed in the alloy.

### 3.2. Texture Evolution of Specimens after One Cycle of MDIF

**Figure 7:**
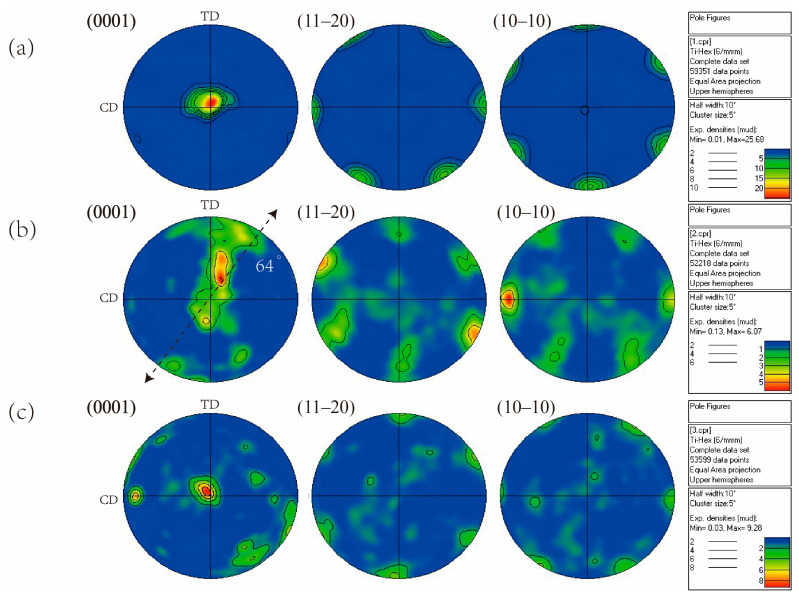
Pole figures of TC4 alloy after one cycle of MDIF: (**a**) one pass; (**b**) two passes; (**c**) three passes.

To obtain insight into the microstructural changes occurring, the (0001), (1120), and (1010) pole figure maps obtained from EBSD are presented in Figure 7, in which CD and TD represent the compression and transverse direction. The polar figure shows that the strongest texture was observed after the first pass of MDIF, and the high-density points in the (0001) pole figure were 25.68. It is noticeable that the (1120) and (1010) pole figures had six points located around the circumference, indicating a typical (0001) <1120> basal texture. After the second pass of deformation, a new texture tilted about 64° to the compression axis was observed in the figure. When one cycle of MDIF was performed, the new grains formed by dynamic recrystallization had a new orientation, weakening the deformation texture.

**Figure 8:**
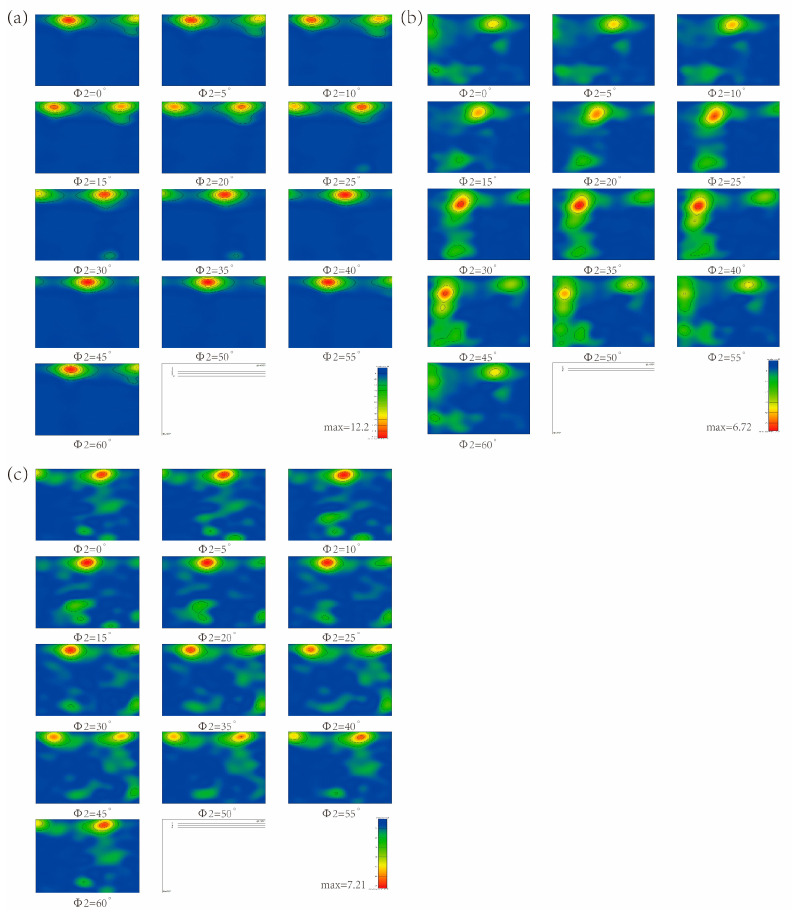
ODF image of TC4 alloy after one cycle of MDIF: (**a**) one pass; (**b**) two passes; (**c**) three passes.

To obtain more information on the type and the degree of orientation density diffusion of the texture, the sections of orientation distribution functions (ODFs) derived from the EBSD images for MDIF TC4 alloy are shown in Figure 8. After one pass of MDIF, the (0002) [21 10] and (0002) [1210] textures were concentrated in the two cross-sections Φ2 = 0° and Φ2 = 30°, as shown in Figure 8a. One can see that the Euler angles of the strongest texture density were (8°, 30°, 0°). Meanwhile, there were also some other textures, such as (0002) [1210], (0002) [21 10], and (2110) [0221]. The Euler angles of these textures were (6°, 90°, 0°), (5°, 3°, 30°), and (85°, 65°, 30°). It can be concluded that, after one pass of MDIF, the type of texture was mainly composed of the basal (0002) [1210] texture. Figure 8b shows that, with the increase in MDIF passes, the texture density was lower than one pass of MDIF. However, the diffuse degree of texture showed an increasing trend. The (0002) [01 10] and (2110) [0112] textures were observed, with Euler angles of (9°, 88°, 30°) and (80°, 25°, 30°). It is noteworthy that the (0002) [1100] texture, with Euler angles of (20°, 30°, 30°), had the strongest density. The type of texture was mainly composed of basal texture (0002) [1010]. After three passes of MDIF, as can be seen from the Figure 8c, the texture was more diffuse than after two passes of MDIF, and the texture density increased. It can be observed that the textures were concentrated in the two cross-sections Φ2 = 10° and Φ2 = 30°. The highest density of texture, which was obtained for (0002) [01 10], occurred at the Euler angles of (8°, 30°, 30°). From the above, it follows that, after one cycle of MDIF, the degree of texture diffusion increased. It should be emphasized that the texture density first decreased and then increased. Although the composition of the texture increased, the main texture type was still the typical (0002) basal texture.

### 3.3. Microstructure of Specimens after Four Cycles of MDIF

**Figure 9:**
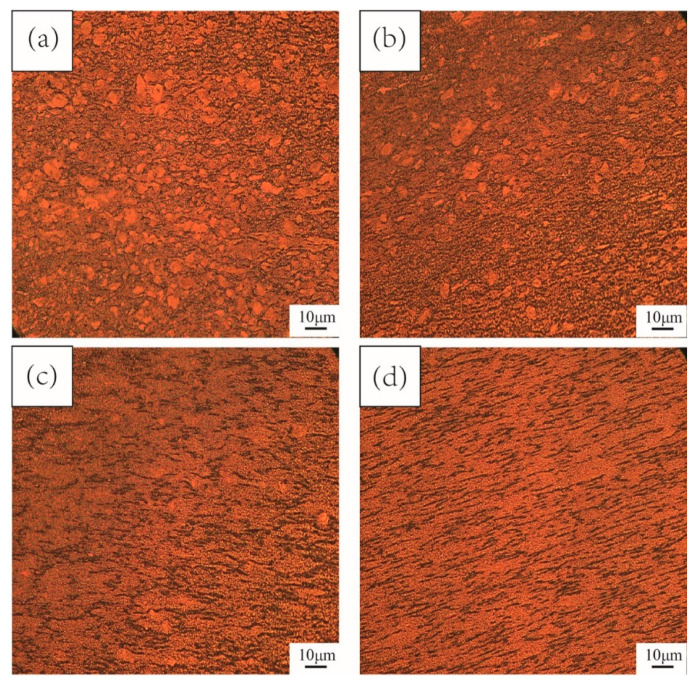
Microstructure of TC4 alloy after MDIF: (**a**) one cycle; (**b**) two cycles; (**c**) three cycles; (**d**) four cycles.

Typical microstructures of the different cycles of MDIF TC4 alloy are presented in Figure 9. It is well known that dynamic recrystallization is an effective way to refine grains [21]. It can be observed that the fine dynamic recrystallized grains were distributed along the elongated grains. In addition, it can also be seen that recrystallization accounted for a small proportion, and the “necklace” structure was not apparent. After two cycles of MDIF, as can be seen from Figure 9b, the thickness of the elongated large grains, surrounded by fine grains, was smaller than in the previous deformation cycle. Moreover, “necklace” structures were generated. The number of large grains decreased with the increase in deformation passes. It should be pointed out that the number of recrystallized grains increased. These recrystallized grains were around and inside the deformed large grains. When four cycles of MDIF were performed, a homogeneous equiaxed grained microstructure with an average grain size of 0.14 μm was achieved. This is quite consistent with the investigation results on TC4 alloy by Li et al. [22].

**Figure 10:**
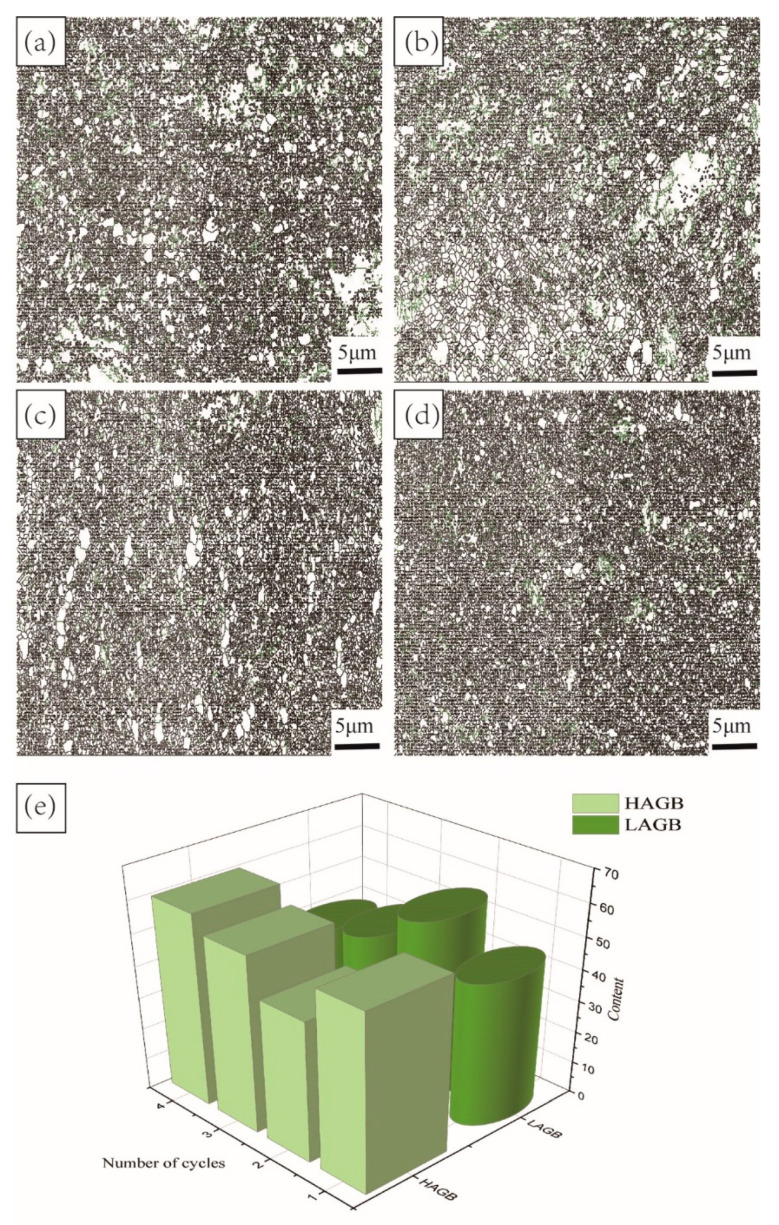
Grain boundary distribution of TC4 alloy after MDIF: (**a**) one cycle; (**b**) two cycles; (**c**) three cycles; (**d**) four cycles; (**e**) variation trend of grain boundary quantity.

Figure 10 shows the grain boundary distribution of TC4 alloy after four cycles of MDIF. In Figure 10, the green lines represent the low-angle grain boundaries (LAGB) with a misorientation angle of 2–15°.The black lines represent the high-angle grain boundaries (HAGB) with a misorientation angle over 15°. Figure 10a shows the grain boundary distribution of TC4 alloy after one cycle of MDIF. According to the statistical data offered by channel 5, the number fraction of LAGB represented by the green line was 45%. In contrast, the number fraction of HAGB was 55%. After two cycles of MDIF, the number fraction of LAGB was 56.2%, and that of HAGB was 43.8%. According to the increase in LAGB, it can be deduced that the dislocations of the alloy increased during the MDIF process. When the alloy was processed by three and four cycles of MDIF, the number fraction of LAGB was 44.8% and 39.8%, while the number fraction of HAGB was 55.2% and 60.2%, respectively. From the above, it follows that, with the increase in deformation cycle (except the first deformation cycle), the content of LAGB decreased, while that of HAGB increased, as shown in Figure 10e. The above research results illustrate the occurrence of dynamic recrystallization in TC4 alloy during the MDIF process.

**Figure 11:**
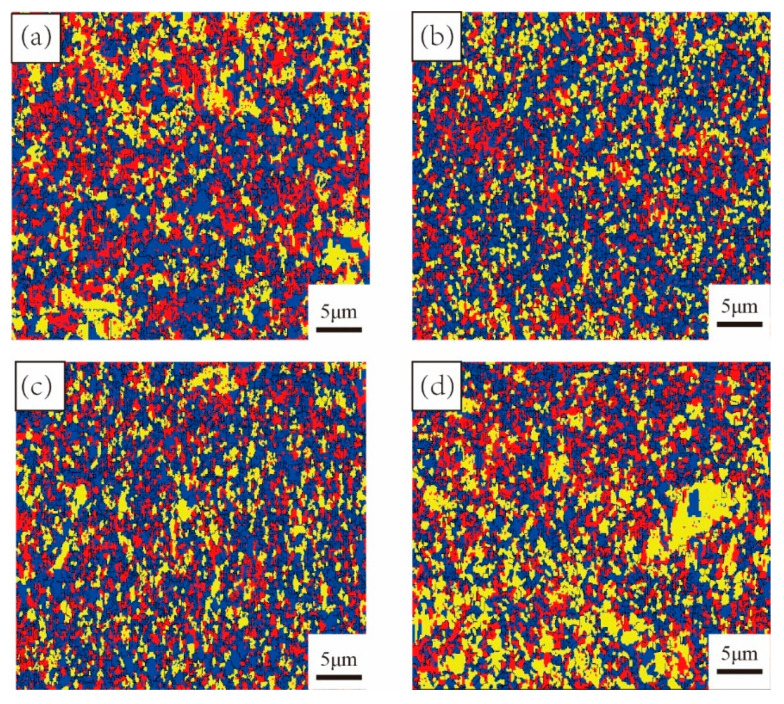
Recrystallization distribution of TC4 alloy after MDIF: (**a**) one cycle; (**b**) two cycles; (**c**) three cycles; (**d**) four cycles.

To distinguish the recrystallized grains and deformed grains, the recrystallization distribution diagram is shown in Figure 11. The deformed grains are represented by red regions. Yellow regions represent the sub-grains. The remaining grains classified as recrystallized are represented by blue regions. As can be summarized from Figure 11, with the process of MDIF, a large number of recrystallization structures and deformation substructures were distributed in the microstructure. A characteristic of MDIF is the constantly changing pressure direction. This results in the intersection of deformation bands. Due to dislocation entanglement [23], the cellular structure changes to sub-grains. With increasing MDIF cycles, the sub-grain changes into a new grain. This result is quite consistent with the investigation results on TC4 alloy by Zhang et al. [17].

**Figure 12:**
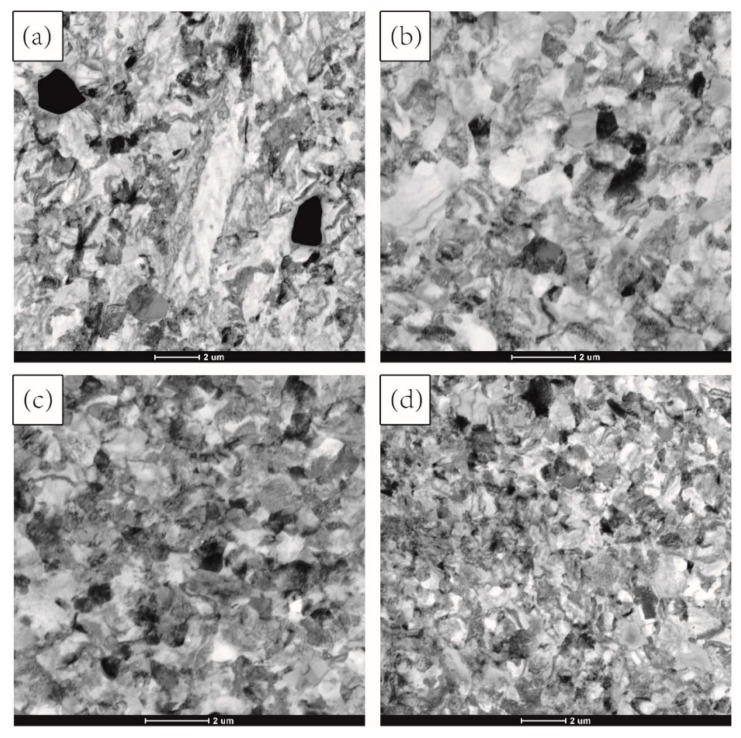
TEM image of TC4 alloy after MDIF: (**a**) one cycle; (**b**) two cycles; (**c**) three cycles; (**d**) four cycles.

To further explore the hot deformation behavior of the alloy, TEM was performed on the investigated samples. Figure 12 shows the TEM bright-field images of samples with different cycles. It is noticeable that, with increasing MDIF cycles, the grains were effectively refined, consistent with the recrystallization distribution of TC4 alloy in Figure 11.

**Figure 13:**
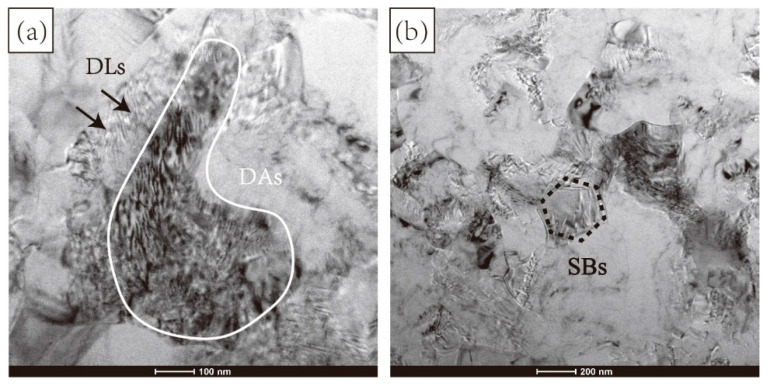
Evolution of dislocation configuration of TC4 alloy after MDIF: (**a**) one cycle; (**b**) four cycles.

A comprehensive study of dislocations was helpful to reveal the grain refinement mechanism of TC4 alloy during MDIF. Figure 13 shows the evolution of dislocations in the microstructure of TC4 alloy after one cycle and four cycles of MDIF. As shown in Figure 13a, dislocations formed dislocation lines due to large strain and changes in the loading direction (as indicated by the white lines). It has been reported that these dislocation lines are caused by cross-slip [24]. These dislocation lines were clustered in high-density dislocation areas (DAs). When the number of deformation passes increased, dislocations gradually formed dislocation walls in the grain. Taking continuous accumulation of strain into account, it was found that the reaction of the dislocation wall was aggravated, and new grains were formed under the action of dynamic recovery, as shown in Figure 13b.

**Figure 14:**
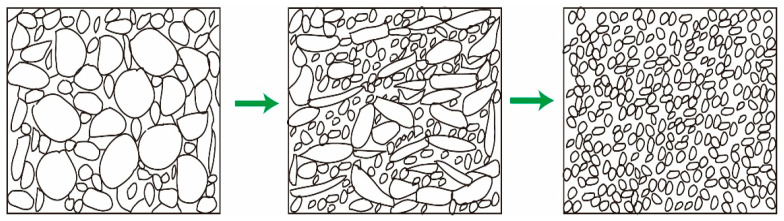
Schematic diagram of recrystallization of TC4 alloy during MDIF process.

We constructed a complete mechanism diagram to describe the nucleation growth of dynamic recrystallization (DRX), as shown in Figure 14. Firstly, under severe deformation, the deformation preferentially expanded in some grains and produced micro deformation bands. Under the alternating change of compression axis, these micro deformation zones intersected. Afterward, the micro deformation bands expanded along a certain path with strain accumulation and led to grain breakage. Finally, the micro deformation zone characterized by severe lattice distortion became the preferential nucleation zone of DRX.

### 3.4. Texture Evolution of Samples after Four Cycles of MDIF

**Figure 15:**
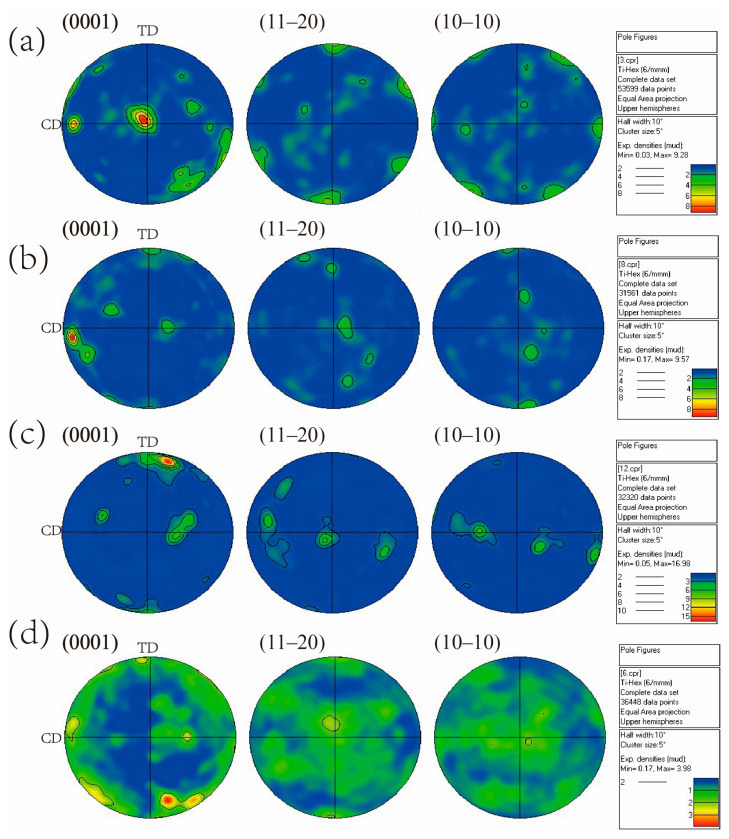
Pole figures of TC4 alloy after MDIF: (**a**) one cycle; (**b**) two cycles; (**c**) three cycles; (**d**) four cycles.

Figure 15 shows the (0001), (1120), and (1010) pole figure maps of TC4 alloy after different cycles of MDIF. The orientation density on the (1120) and (1010) pole figures was weak, while the orientation density on the (0001) pole figure was strong. According to the above analysis in Figure 8, after one cycle of MDIF, the texture type in TC4 alloy was the (0002) [1010] basal texture. When two cycles of MDIF were performed, the orientation density of the (0001) pole figure was concentrated at a 15° angle to the compression direction. It can be noted that the (1120) <1010> texture was observed, and the strength of texture was stronger than the previous cycle of MDIF, as shown in Figure 15b. After three cycles of MDIF, as shown in Figure 15c, the orientation density of the (0001) pole figure was concentrated at an 8°angle to the compression direction. The (1120) <0001> texture was formed. After four cycles of MDIF, it can be seen from Figure 15d that the type of texture was mainly composed of (1010) <1210>. Additionally, the density of the texture decreased, and the diffusion of texture became more obvious.

**Figure 16:**
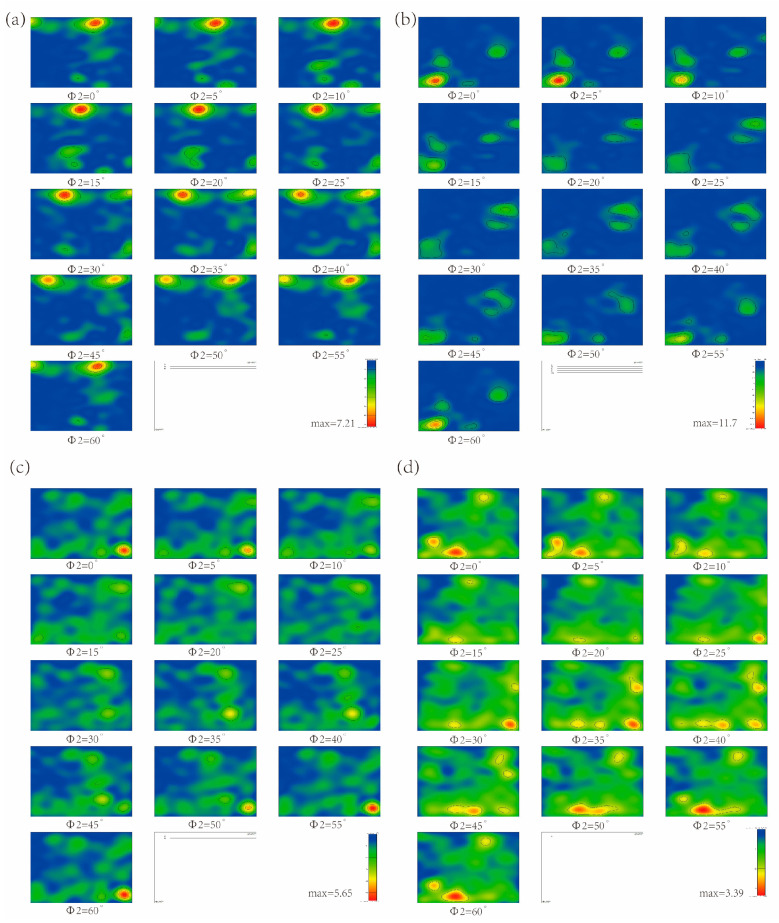
ODF image of TC4 alloy after MDIF: (**a**) one cycle; (**b**) two cycles; (**c**) three cycles; (**d**) four cycles.

Figure 16 shows the sections of orientation distribution functions (ODF) images of TC4 alloy to determine the type and degree of orientation density diffusion of the texture. The analysis after one cycle of MDIF was already discussed in Figure 8. Here, we focus on the subsequent deformation cycles. As seen from Figure 16b, the texture was concentrated in the two cross-sections Φ2 = 0° and Φ2 = 30°. The Euler angles of texture were (82°, 15°, 0°), (60°, 20°, 0°), (46°, 70°, 0°), (85°, 45°, 0°), and (27°, 78°, 30°), with the corresponding textures of (1010)[0001], (1011)[21 13], (1012)[1100], (1010)[1212], and (0002)[0110], respectively. One can see that the density of texture was strongest at the Euler angles of (82°, 15°, 0°), indicating that the type of texture was mainly composed of (1010) <0001> after two cycles MDIF. In addition, the texture density increased, and the diffusion degree decreased compared with the one cycle MDIF. After three cycles of MDIF, as shown in Figure 16c, the texture was concentrated in the two cross-sections Φ2 = 0° and Φ2 = 5°. The Euler angles of texture were (70°, 62°, 0°), (17°, 88°, 0°), (80°, 82°, 0°), and (82°, 8°, 5°), with the corresponding textures of (1011)[1211], (0002)[1210], (1010)[1210], and (1010)[0001], respectively. It is obvious that the density of texture was strongest at the Euler angles of (80°,82°,0°), indicating that the type of texture was mainly composed of (1010)[1210]. It is noticeable that the texture density decreased and the diffusion degree increased compared with the two cycles of MDIF. After four cycles of MDIF, as shown in Figure 16d, the texture was concentrated in the two cross-sections Φ2 = 0° and Φ2 = 30°. The Euler angles of texture were (13°, 60°, 0°), (68°, 14°, 0°), (82°, 33°, 0°), (82°, 82°,30°), and (35°, 87°, 30°), with the corresponding textures of (0002)[1100], (1011)[1102], (1010)[1102], (2110)[0110], and (2115)[0110], respectively. The Euler angles of the strongest texture density were (82°, 33°, 0°), indicating that the type of texture was mainly composed of (1010) [1102]. Furthermore, the texture density decreased, and the diffusion degree increased after four cycles of MDIF.

### 3.5. Mechanical Properties

**Figure 17:**
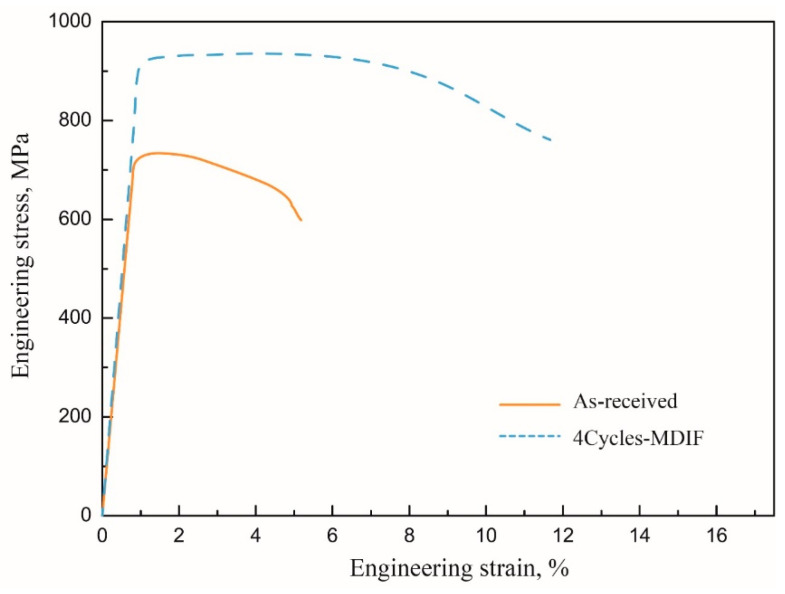
Tensile engineering stress–strain curves of TC4 alloy.

The engineering stress–strain diagrams obtained by tensile testing at room temperature for MDIF-processed materials are shown in Figure 17. It can be clearly seen that the tensile properties of the as-received TC4 alloy were inferior. After four cycles of MDIF, the yield strength (YS) and the ultimate tensile strength (UTS) increased from 742 MPa and 747 MPa to 900 MPa and 921 MPa, respectively. Meanwhile, the elongation increased from 3.9% to 12.1%. It can be concluded that, with the increase in multidirectional compression cycle, the grain of the TC4 alloy became smaller, and the tensile strength and elongation of the alloy improved. It has been widely accepted that the strength properties of polycrystalline materials depend on their grain size [25]. The mechanism of mechanical property enhancement is considered to be grain boundary strengthening. Sabirov et al. [26] reported that grain boundaries generally act as obstacles to dislocations passing from one grain into another, resulting in the pile up of dislocations near the grain boundaries. Moreover, the strain inside and near the grain boundary of the finer grains varies little, and the deformation is more uniform, which leads to a larger elongation and reduction of a section.

**Figure 18:**
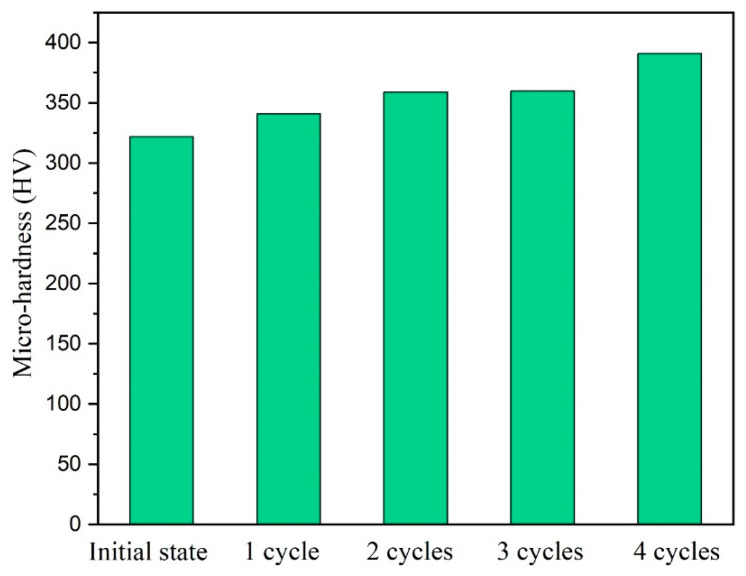
Micro hardness of the sample before and after each cycle of MDIF.

Figure 18 shows the hardness values of the sample before and after each cycle of MDIF. It can be concluded that the hardness of the alloy increased with the increase in MDIF cycles. For example, after two cycles of deformation, the hardness value of the alloy was 359 (HV), which increased by 11% compared to the undeformed alloy. This phenomenon can be attributed to the increase in dislocation density [27], which can effectively resist external deformation.

## 4. Conclusions

The microstructure and mechanical behavior of TC4 alloy processed by MDIF were examined. The variation of the tensile yield strength was correlated to the change in microstructure during SPD. The following conclusions can be drawn:

(1) After one cycle of MDIF, the average grain size of the α-phase was refined from 4.8 μm to 0.68 μm. The deformation mechanism was mainly dislocation slip. When four cycles of MDIF were performed, a homogeneous equiaxed grained microstructure with an average grain size of 0.14 μm was achieved.

(2) After one cycle of MDIF, the texture density first decreased and then increased. The diffusion degree increased. The type of texture was mainly composed of (0002) <1010> and (0002) <1120>. The Euler angles of the strongest texture density were (8°, 30°, 0°). With the increase in MDIF cycles, it was found that the density of texture decreased, and the degree of dispersion of texture increased. Furthermore, the non-basal texture (1010) <1102> was observed, with Euler angles of (82°, 33°, 0°).

(3) After four cycles of MDIF, the yield strength and the ultimate tensile strength increased from 742 MPa and 747 MPa to 900 MPa and 921 MPa, respectively. Meanwhile, the elongation increased from 3.9% to 12.1%. The main reason for the improvement of mechanical properties was attributed to grain refinement. Moreover, the hardness of the alloy increased with the increase in MDIF cycles. This can be attributed to the increase in dislocation density.

## Figures and Tables

**Table 1:** Chemical composition of as-cast Ti-6Al-4V alloy (wt.%).

	Ti	Al	V	Fe	C	H	O	N
wt.%	Bal.	6.45	4.21	0.2	0.06	0.008	0.17	0.01

## Data Availability

The data will be made available upon request.
